# A pore-forming protein implements VLR-activated complement cytotoxicity in lamprey

**DOI:** 10.1038/celldisc.2017.33

**Published:** 2017-09-19

**Authors:** Fenfang Wu, Bo Feng, Yong Ren, Di Wu, Yue Chen, Shengfeng Huang, Shangwu Chen, Anlong Xu

**Affiliations:** 1 State Key Laboratory of Biocontrol, Guangdong Province Key Laboratory for Pharmaceutical Functional Genes, College of Life Sciences, Sun Yat-Sen University, Guangzhou, Guangdong, China; 2 School of Life Sciences, Beijing University of Chinese Medicine, Beijing, China

**Keywords:** complement, cytotoxicity, lamprey, pore-forming protein, VLR

## Abstract

Lamprey is a basal vertebrate with a unique adaptive immune system, which uses variable lymphocyte receptors (VLRs) for antigen recognition. Our previous study has shown that lamprey possessed a distinctive complement pathway activated by VLR. In this study, we identified a natterin family member–lamprey pore-forming protein (LPFP) with a jacalin-like lectin domain and an aerolysin-like pore-forming domain. LPFP had a high affinity with mannan and could form oligomer in the presence of mannan. LPFP could deposit on the surface of target cells, form pore-like complex resembling a wheel with hub and spokes, and mediate powerful cytotoxicity on target cells. These pore-forming proteins along with VLRs and complement molecules were essential for the specific cytotoxicity against exogenous pathogens and tumor cells. This unique cytotoxicity implemented by LPFP might emerge before or in parallel with the IgG-based classical complement lytic pathway completed by polyC9.

## Introduction

Complement is an essential component of the immune system that eliminates foreign pathogens mainly by forming a pore-like membrane attack complex (MAC) [1, 2] on the target cell membrane in mammals. In jawed vertebrates, the classical complement pathway is activated by antigen-antibody complex binding to complement C1q. Lampreys, the most basal jawless vertebrates, have a unique adaptive immune system consisting of variable lymphocyte receptors (VLRs) instead of IgG or IgM, TCR and BCR of jawed vertebrates [3, 4]. Therefore, it has long been questioned whether lamprey possesses a classical complement pathway. In previous study, we have shown that complement-dependent cytotoxicity mediated by VLR in lamprey is an alternative of the classical activation pathway of mammalian [5]. Failing to find complement C6 to C9 [6] in lamprey has complicated the unambiguous identification of the complement terminal lytic molecules. It remains unknown as to what key effector molecules may implement the lysis of target cells in the final step of the cytotoxicity.

Here, we identified a lamprey pore-forming protein, named as LPFP, depositing on the membrane of target cells and killing them. Thus, the VLRs functionally link to complement components C1q, C3 [5, 7–9] and LPFP reported here acts as a defensive weapon against exogenous pathogens or mutant cells. LPFP may serve the key roles to understand the defense mechanism of this basal vertebrate and the origin and evolution of vertebrate adaptive immunity.

## Results

### Powerful cytotoxicity of lamprey antisera

**Figure 1:**
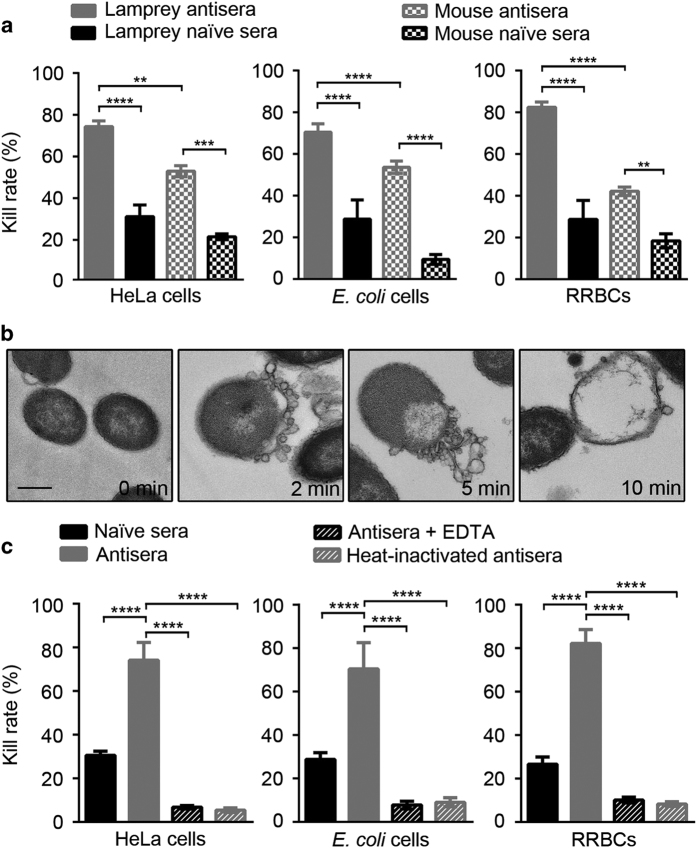
Cytotoxicity characterization of lamprey antisera. (**a**) Hela cells, *E.coli* cells and rabbit red blood cells (RRBCs) were killed by lamprey sera or mouse sera. The cytotoxicity of different sera was evaluated by the kill rate of target cells. (**b**) Effects on *E. coli* after exposure to lamprey antisera. Samples were treated by 10% lamprey antisera for 0, 2, 5, or 10 min, respectively. Scale bar, 500 nm. (**c**) Lamprey antisera were treated with EDTA or heated at 56 °C for 30 min. The cytotoxicity of lamprey sera before and after treatment was evaluated by the kill rate of target cells. Data are the means±s.d. from three independent experiments. Statistical differences were determined by one-way ANOVA and Tukey’s multiple comparison test. **P<0.01, ***P<0.001 and ****P<0.0001.

In previous study we showed that lamprey antisera had a strong specific complement-dependent cytotoxicity to target cells, which was mediated by VLR along with C1q and C3 [5]. This complement activation pathway was the alternative classical pathway of jawed vertebrates. In this study, we compared the cytolytic effects of lamprey antisera with mouse antisera. About 80% of the target cells were destroyed by the lamprey antisera, whereas only 40–50% cells were killed by the mouse antisera (Figure 1a). The mechanism by which the target cells were destroyed was investigated by examining the lamprey antiserum-treated *Escherichia coli* (*E. coli*) cells for different time periods via transmission electron microscopy (TEM). The cells underwent obvious necrosis, with small bubbles on the cell surface, loss of membrane integrity, and leakage of cell contents through the cell membranes, eventually resulting in complete cell lysis (Figure 1b). These bubbles were also observed in HeLa cells killed by lamprey antisera, but not by mouse antisera (Supplementary Figure S1). These results suggested that there was a different killing mechanism between lamprey and mouse antisera. Comparison of the two antisera showed that the lamprey antisera had a more powerful complement-dependent cytolytic effect. The cytotoxicity of lamprey antisera obviously declined when heat-inactivated or Ca^2+^ and Mg^2+^ were chelated by EDTA (Figure 1c), which were the important features of complement. The naive sera also showed some degree of cytolytic effect on these cells, possibly because of other complement activation pathway.

### Discovery of lamprey pore-forming protein

**Figure 2:**
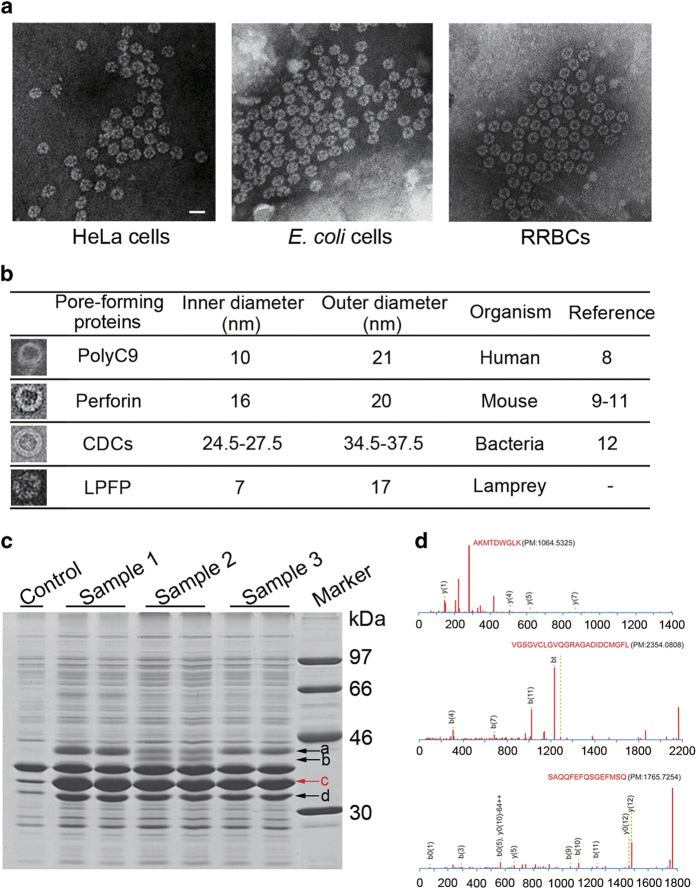
Discovery of a pore-like complex LPFP in lamprey. (**a**) TEM of membrane protein from RRBC, *E. coli* and HeLa cells killed by lamprey antisera. Scale bar, 20 nm. (**b**) Comparison of pore-like complexes in selected species. (**c**) SDS–PAGE of membrane proteins extracted from *E. coli* cells treated with lamprey antisera (samples 1, 2 and 3). The membrane proteins extracted from *E. coli* cells treated with PBS were used as control. Four bands a-d were identified by mass spectrometry. (**d**) Three peptides obtained from mass spectrometry of band c were corresponding to an uncharacterized protein with UniProtKB/TrEMBL accession number of S4RHQ3.

Since the complement C6-C9 have not been found in lamprey [6], it has long been questioned whether lamprey possesses a pore-forming protein analogous to C9 that can cause invading pathogens to lyse. To search and identify the potential pore-forming proteins, we extracted the membrane proteins using ultracentrifugation from *E. coli*, HeLa cells, and rabbit red blood cells (RRBC) before or after being treated with the corresponding lamprey antisera. Then, we examined the extracted proteins by TEM after negative staining with uranyl formate. Fortunately, many pore-like complexes that resembled hub-and-spoke wheel structure with a 7-nm inner diameter and 17-nm outer diameter were observed on the membrane of all three types of cells only after treated with lamprey antiserum (Figure 2a and Supplementary Figure S2). Comparison with other well-known pore-like complexes, we found that lamprey pore-like complexes were unique both in size and shape. The membrane attacking complex (MAC), composed of polyC9, is a ring-shaped pore with inner diameter of 10 nm and outer diameter of 21 nm [10]. Perforin has structural and functional similarities to polyC9 and it can polymerize into polyC9-like transmembrane pore with inner and outer diameters of 16 and 20 nm, respectively [11–13]. The cholesterol-dependent cytolysins (CDCs) disrupt the membrane of target cell by forming pores with outer diameter of 34.5–37.5 nm and inner diameter of 24.5–27.5 nm [14] (Figure 2b). These data demonstrated that lamprey had a unique effector molecule to implement the lysis of the target cells.

To further identify the pore-forming complex, the extracted proteins from *E. coli* treated with or without lamprey antisera were subjected to SDS–PAGE analysis. Compared with the normal *E. coli* membrane protein, there were four extra bands to be found in the three treated samples (Figure 2c, bands a-d). In order to identify these proteins, each band was cut and digested by trypsin, and their amino acid sequences were determined by mass spectrometry (MS). We obtained a number of sequences from MS which match with serum lectin, VLR, C3, intelectin and many other known and unknown proteins through comparison with the sea lamprey transcriptome. After searching in http://www.ebi.ac.uk/services, we found that three peptides (Figure 2d) corresponding to an uncharacterized protein with UniProtKB/TrEMBL accession number of S4RHQ3 which had a putative pore-forming domain. We further blasted at NCBI and found the uncharacterized protein S4RHQ3 was homologous to the natterin-like protein (GenBank: AFX60113.1) [15] of *Lampetra japonica*. Natterin protein was first found in the venom of *Thalassophryne nattereri* as a kininogenase, and was named after the species name [16].

### Structure and expression pattern of lamprey pore-forming protein

**Figure 3:**
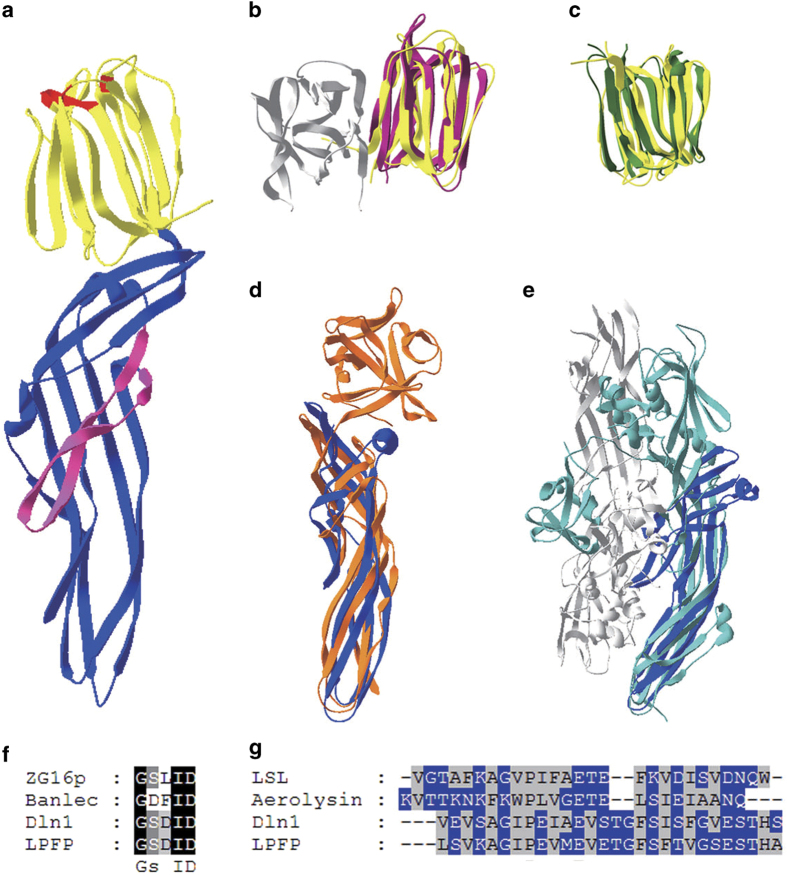
Structure prediction of LPFP. (**a**) Tertiary structure of LPFP predicted by Phyre2 based on the template of Dln1 (PDB 4ZNO). The structure is composed of a jacalin-like lectin domain (yellow) and an aerolysin-like pore-forming domain (blue). The conserved sugar-binding site in the lectin domain is colored in red, and the pre-stem domain of pore-forming domain is colored in pink. (**b**) Structure comparison of LPFP-lectin domain (yellow) with banana lectin (purple, PDB 3MIT). Another subunit of banana lectin is colored in gray. (**c**) Structure comparison of LPFP-lectin domain (yellow) with human ZG16p lectin (green, PDB 3VY7). (**d**) Structure comparison of LPFP-pore-forming domain (blue) with LSL (orange, PDB 1W3A). (**e**) Structure comparison of LPFP-pore-forming domain (blue) with proaerolysin (light blue, PDB 1PRE). Another subunit of proaerolysin is colored in gray. (**f**) Conserved GXXXD motif in different jacalin-like lectins. (**g**) The sequences of pre-stem domains of different aerolysin-like pore-forming proteins, the hydrophilic (blue) and hydrophobic (gray) residues are arranged alternately.

According to the sequence of natterin-like protein of *Lampetra japonica*, we firstly obtained the full-length cDNA sequence of natterin-like protein in *Lampetra morii* by performing 3′ rapid amplification of cDNA ends (3′-RACE) and 5′-RACE. The full-length cDNA was 1 189 bp (Supplementary Figure S3) with a 942-bp open reading frame encoding a 313-amino acid protein (Supplementary Figure S4). Tertiary structure was predicted by Phyre2 Protein Fold Recognition Server (http://www.sbg.bio.ic.ac.uk/phyre2/html/page.cgi?id=index) based on the templete of zebrafish Dln1 [17] (PDB:4ZNO) and we found that it was composed of a jacalin-like lectin domain at the N-terminus and an aerolysin-like pore-forming domain at the C-terminus (Figure 3a). On the basis of its putative pore-forming domain, the natterin-like protein in *Lampetra morii* was named lamprey pore-forming protein (LPFP). Dln1 was also a natterin-like protein with pore formation features which had high sequence homology with LPFP (Supplementary Figure S5). Furthermore, structural comparison with DALI server (http://ekhidna.biocenter.helsinki.fi/dali_server/start) also showed that N-terminal of LPFP was similar to jacalin-like banana lectin [18] (PDB 3MIT, Figure 3b) and human ZG16p lectin [19] (PDB 3VY7, Figure 3c), and the C-terminal was close to aerolysin pore-forming proteins LSL [20] (PDB 1W3A, Figure 3d) and proaerolysin [21] (PDB 1PRE, Figure 3e). The C-terminal of LPFP has a pre-stem loop, which was identified as the transmembrane region of aerolysin family. In pre-stem of LPFP, the hydrophilic and hydrophobic residues are arranged alternately, which was well conserved in aerolysin family (Figure 3g). Although there was no direct result demonstrating that Dln1 could kill cells, findings on the structure and characters provide insights into a putative immune defense molecule in bony fish and lamprey. To further identify the similarity and genetic relationship of LPFP with other natterins, lectin proteins or pore-forming proteins, we performed homology alignment and phylogenetic analysis. LPFP was closer to the natterin in fish species than other species (Supplementary Figure S6a) but far from other lectins (Supplementary Figure S6b) or pore-forming proteins (Supplementary Figure S6c).

**Figure 4:**
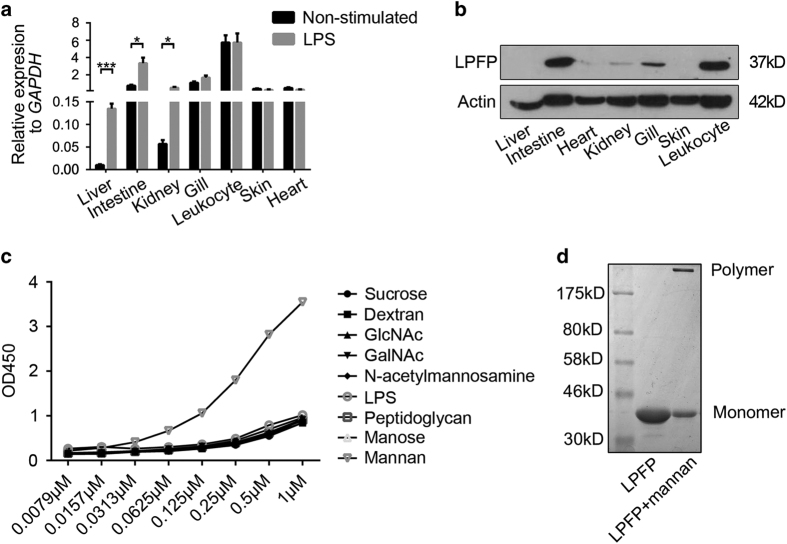
Features of lamprey pore-forming protein. (**a**) Expression levels of LPFP mRNAs in various tissues before and after stimulation with LPS. The relative expression is compared with GAPDH. Data are the means±s.d. from three independent experiments. Comparisons were performed with *t*-tests (two groups). *P<0.05, **P<0.01, ***P<0.001. (**b**) Expression levels of LPFP in various tissues by western blot assay. (**c**) Sugar-binding assay of LPFP with different carbohydrates by ELISA. Eight carbohydrates coated on the 96-well ELISA plate were incubated with LPFP by 0.5-log dilutions from 1 to 0.0079 μm. (**d**) LPFP was incubated with mannan for 1 h. Then the mixture and LPFP was detected by SDS–PAGE respectively. The bands corresponding to the monomer or oligomer were marked.

Gene expression analysis showed that *LPFP* mRNA was present in many tissues, and most abundant in leukocytes, intestine, and gill (Figure 4a). LPFP protein expression level in tissues was identical with mRNA expression (Figure 4b). Upon stimulation with lipopolysaccharides (LPS), the levels of LPFP mRNA in the liver, kidney and intestine increased significantly (Figure 4a), suggesting that LPFP may be involved in lamprey immune defense.

### Sugar binding and oligomerization of LPFP

The jacalin-like lectin domain is a mannose-binding lectin domain. The main residues (Gly15, Arg87, Gly131, Ser132, Asp133 and Asp135) for sugar recognition and binding in Dln1 were conserved in LPFP (Supplementary Figure S5). Similar to Dln1 and other lectins [18], LPFP also has a G^131^XXXD^135^ motif for sugar binding (Figure 3f). We detected the sugar binding specificity of LPFP to different sugars by ELISA and found that LPFP had a high binding affinity with yeast mannan rather than manose and other carbohydrates (Figure 4c). Mannan is a cell wall polysaccharide found in yeasts, implying a potential defense role of LPFP. But previous study reported Dln1 specifically adheres to yeast cells but could not trigger cell death [17]. These provide a possibility that natterin-like protein plays a role in a more complex mechanism.

The most important function of pore-forming proteins is to oligomerize and form pores on the membrane of invading cells leading to cell lysis. Then, we detected the oligomerization of LPFP. SDS–PAGE showed that LPFP could form oligomers in the presence of mannan (Figure 4d). We tried to find the pore-like structure formed by these oligomers under TEM, but failed. A possible reason is that the pore formation process requires certain conditions, like membrane structure or other receptors.

### Cytotoxicity of LPFP from lamprey antisera

**Figure 5:**
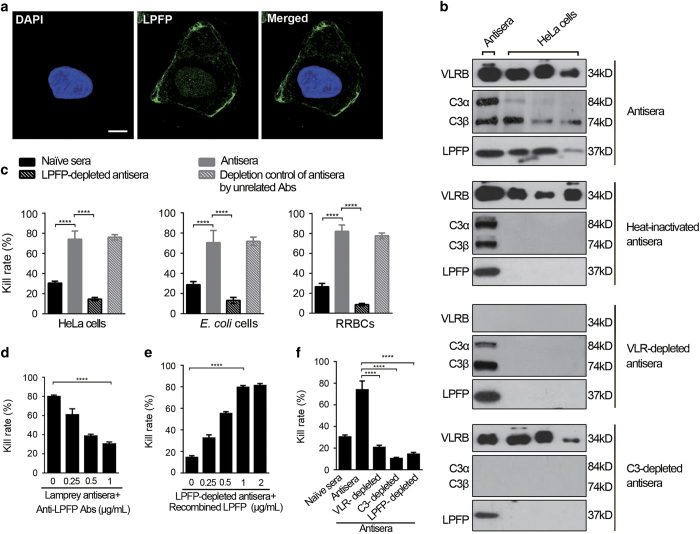
Function analysis of LPFP in cytotoxicity induced by lamprey complement. (**a**) Lamprey LPFP deposited on the surface of HeLa cells treated with lamprey antisera. (**b**) Effect of VLR or C3 on the deposition of LPFP on the surface of HeLa cells treated with lamprey antisera. Antisera were from three lampreys immunized with HeLa cells. (**c**) Effects of LPFP-depletion on the cytotoxicity exerted on target cells by lamprey antisera. (**d**) Dose-dependent effect of anti-LPFP antibody on the cytotoxicity of lamprey antisera. Lamprey antisera directed against HeLa cells was treated with the indicated concentrations of anti-LPFP antibody, and the residual cytotoxicity of the treated antisera was evaluated based on the killing rates of HeLa cells. (**e**) Effect of recombinant LPFP on the cytotoxicity exerted on HeLa cells by LPFP-depleted lamprey antisera. (**f**) Contribution of VLR, C3, and LPFP to the cytotoxic effect exerted by lamprey antisera against HeLa cells. Data are the means±s.d.s from three independent experiments. Statistical differences are determined by one-way ANOVA and Tukey’s multiple comparison test (**c**–**f**). ****P<0.0001.

Our previous study has shown that lamprey possesses a distinctive complement pathway activated by VLR and such pathway eventually induces specific cell lysis through C3 activation [5]. The recombinant LPFP was expressed in *E.coli* and rabbit anti-LPFP polyclonal antibody was prepared to study the function of LPFP (Supplementary Figure S7). However, the molecular mechanism underlying the terminal lysis of target cell is unknown. In the present study, we found that LPFP could deposit on the surface of HeLa cells treated with lamprey antisera (Figure 5a). We inactivated the complement with heat inactivation or by removing key complement activation molecules in the serum to verify whether the deposition of LPFP on the target cell surface was complement- dependent or not. Upon heat-inactivation or depletion of VLRB or C3, the deposition of LPFP on HeLa cells was almost completely suppressed (Figure 5b). In addition, when LPFP was depleted by rabbit anti-LPFP polyclonal antibody, the cytotoxicity of the LPFP-depleted antisera was significantly reduced (Figure 5c), and such reduction was in a dose-dependent manner (Figure 5d). Cytotoxicity was restored by adding back recombinant LPFP, which was also in a dose-dependent way (Figure 5e). Thus, in addition to VLR and C3, LPFP was also found to be essential for the VLR-mediated complement activation pathway in lamprey (Figure 5f).

## Discussion

Complement classical activation pathway is an important bridge connecting innate immunity and adaptive immunity [22]. This pathway is activated by antigen-antibody complex and is thought to emerge only in jawed vertebrates. However, since there is no antibody in the ancient jawless vertebrate lamprey, complement activation pathway is instead activated by VLRs as unique adaptive immunity. In jawed vertebrates, complement lytic pathway is completed by MAC, a pore complex formed by C6–C9. However, the counterpart of C6–C9 has not been identified in lamprey. Thus, whether the complement cytotoxicity of lamprey is also implemented by a pore complex like MAC becomes an intriguing question.

Our previous study showed that VLRB-antigen complex could bind with C1q to mediate an alternative classical pathway in lamprey and trigger a complement-dependent cytotoxic effect. Here, we provide extensive evidences that LPFP, a pore-forming protein from lamprey, is the terminal lytic molecule and implements VLR-activated complement cytotoxicity against target cells. Our findings suggest that this inter-connection of innate and adaptive immunity dates back basal jawless vertebrates, some 500 million years ago.

In lamprey, the cytotoxic effect is stronger than mammals. Under the electron microscope, many wheel-like porins were found in the membrane protein of target cells treated by lamprey antisera. After comparisons, we found this porin was not identical to the pore-form proteins of other species either in shape or size demonstrating that there was a new pore-forming protein to implement lamprey complement lysis. Furthermore, mass spectrometric analysis showed that LPFP was a natterin family protein.

Tertiary structure prediction and structure comparison both revealed that LPFP was composed of a jacalin-like lectin domain in the N-terminal and an aerolysin-like pore-forming domain in the C-terminal. This structure was almost the same with Dln1, a natterin-like protein found in zebrafish, and had a high homology with LPFP. Dln1 was identified to be a putative immune defence molecule by forming pore-like structure like aerolysin [17].

Though Dln1 could specifically adhere to yeast cells, it could not trigger yeast death directly [17]. In our study, for the first time, we found that LPFP could lyse target cells via VLR-mediated and complement-dependent cytotoxicity.

The results indicated that the deposition of LPFP on the target cell surface was dependent on the VLRs and complement system. When LPFPs were neutralized by the specific antibody from the antisera, the complement cytolytic ability was almost completely inhibited. Thus, this study demonstrated that lamprey possessed a previously unrecognized VLR-mediated and complement-dependent cytotoxicity, in which LPFP functions as the key component that initiates the final step of the lytic effect. As an alternative to the classical pathway of complement system, VLR-mediated complement activation pathway probably emerged in primitive jawless vertebrates.

**Figure 6:**
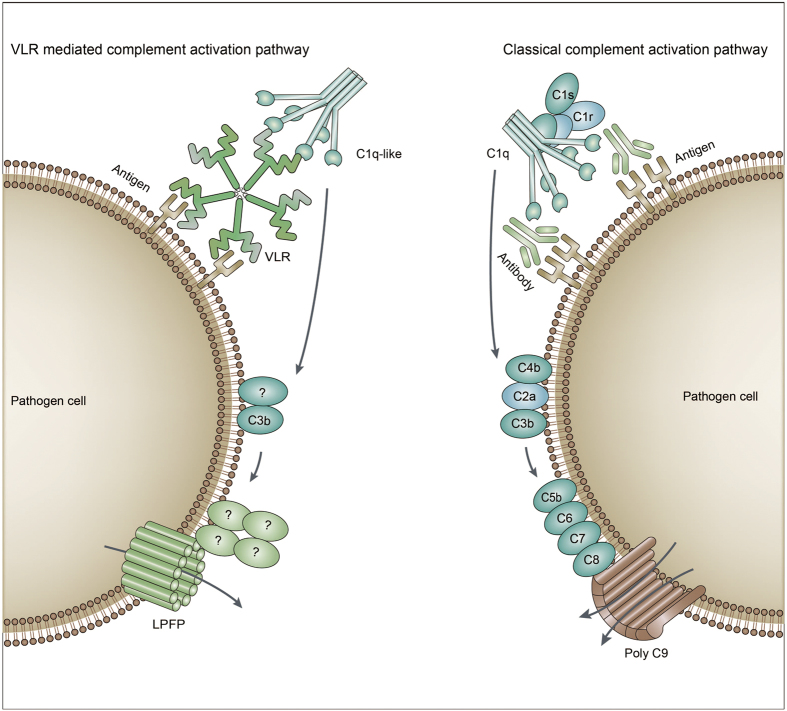
Comparison of the complement activation pathway of jawed and jawless vertebrates. In jawed vertebrates, the classical activation pathway of the complement system is triggered by antigen-antibody complex. The binding of complement C1 to the Fc region of the antibody initiates the complement activation pathway, and C4, C2 and C3 are then successively cleaved and activated. Finally, a subunit composition of C5b-C6-C7-C8-polyC9 forms the MAC that initiates the lysis of the target cells. In jawless vertebrates, the VLR-mediated complement pathway is triggered by antigen-VLR complex and C1q molecules. During this complement activation process, C3 is cleaved and activated. Finally, LPFP is recruited to form a pore complex on the membrane of target cells, resulting in the target cell lysis. Together, the two parallel complement pathways achieve similar cytotoxicity.

In conclusion, jawed vertebrates possess an adaptive immune system that uses V(D)J recombination to create antibodies that are able to recognize antigens [23], while jawless vertebrates employ genetic recombination of *VLR* gene [3, 4]. In jawed vertebrates, the classical activation pathway of the complement system is triggered by antigen-antibody complex. The binding of the C1 component to the Fc region of the antibody initiates the pathway, and C4, C2, and C3 are then successively cleaved and activated [24]. Finally, a subunit composition of C5b-C6-C7-C8-polyC9 forms the MAC that initiates lysis of the target cells [25]. In jawless vertebrates, the VLR-mediated complement pathway is triggered by antigen-VLR complex and C1q molecules. Finally, LPFP is recruited to form a pore complex on the membrane of target cells, resulting in cell lysis. During this process, C3 is cleaved and activated [5]. Together, the two parallel complement pathways achieve similar cytotoxicity (Figure 6). The newly identified pore-like structures containing LPFP on the membranes of the lysed cells were functionally similar to the MAC of mammalian complement. It appears that lamprey defends against the invading pathogens and mutated cells through a powerful immune system including VLR, LPFP, and complement components. Further biochemical, structural, and functional analyses of LPFP will provide additional insights into the origins of complement terminal lysis mechanism and vertebrate adaptive immunity.

## Materials and methods

### Animals, bacterial strains and cells

Adult *Lampetra morii* were captured from the Songhua River in Northeast China and maintained in laboratory environment. The water temperature was kept at 18 °C. *E. coli* (DH5α), HeLa cells and RRBCs were maintained in our laboratory.

### Preparation of lamprey and mouse antisera

Adult lampreys (23–29 g, 23–28 cm) of 20 were separated into four groups, and each group animals were respectively inoculated with 10^8^ *E. coli*, 10^7^ RRBCs, 10^6^ HeLa cells, or 100 μl 0.9% NaCl (control) via four intraperitoneal injections at 10 day intervals. RRBCs and HeLa cells were injected as live cells, while *E. coli* was inactivated by pasteurization at 60 °C for 30 min prior to injection. Five days after the fourth injection, blood was collected from tail-severed lampreys and centrifuged at 4 500 *g* for 10 min. The supernatant was collected and used as antisera. BALB/c (26–28 g) mouse antisera were also produced via four intraperitoneal injections at 14 day intervals by respective inoculating with 10^8^ *E. coli*, 10^7^ RRBCs, 10^6^ HeLa cells and 100 μl 0.9% NaCl as control.

### Determination of cytolytic effects of lamprey antisera

The cytolytic effects assays of lamprey and mouse antisera against HeLa cells, RRBCs and *E. coli* DH5α were as described previously [5]. The kill rate of HeLa cells and RRBCs was evaluated according to the following method:

$$\begin{array}{c} \mathrm{Kill}\;\text{rare}(\%)=\\ \frac{\text{The}\;\text{initial}\;\text{cell}\;\text{number}-\text{number}\;\text{of}\;\text{surviving}\;\text{cells}\;\text{treated}\;\text{by}\;\text{antisera}}{\text{The}\;\text{initial}\;\text{cell}\;\text{number}}\\ \times 100\%. \end{array}$$

The kill rate of *E. coli* was evaluated according to the following method:

$$\begin{array}{c} \mathrm{Kill}\;\text{rare}(\%)=\\ \frac{\text{Number}\;\text{of}\;\text{clonal}\;\text{formation}\;\text{unit}\;\text{CFU}(\text{time}\;\text{zero})-\text{Number}\;\text{of}\;\text{CFU}\;(\text{14}\;\text{h}\;\text{later})}{\text{Number}\;\text{of}\;\text{CFU}\;(\text{time}\;\text{zero})}\\ \times 100\%. \end{array}$$

### Cytolytic effects analysis of lamprey antisera to *E. coli* by electron microscopic

*E. coli* cells were treated with lamprey antisera at 4 °C for 0, 2, 5 and 10 min, respectively. Subsequently, cells were fixed with 2.5% glutaraldehyde solution in 100 mm sodium phosphate buffer (pH 7.2) at room temperature. After washing with 100 mm sodium phosphate, the cells were further fixed with 1% (w/v) osmium tetroxide in phosphate buffer at 4 °C for 2 h. The cells were then dehydrated successively in 70, 80, 90 and 100% ethanol; transferred into propylene oxide; and embedded in Epon812. Ultrathin sections were cut with a Leica EM UC6 ultramicrotome (Wetzlar, Germany) and mounted on a formvar-coated brass grid. The sections were stained with 2% uranyl acetate (w/v) in 70% methanol (v/v) and 0.5% lead citrate. Observations and image recording of the cells were performed with a JEM-2000EX TEM.

### Extraction and negative staining of membrane constituents of cells observed by TEM

RRBCs, *E. coli* and HeLa cells were pre-treated for 30 min at 4 °C by respectively antisera. Samples were washed twice in 10 mm PBS and then subjected to lysis by sonication instrument with the power of 300w on the ice. The total time of ultrasound broken was 10 min, with 3 s on and 3 s off. Crude outer membranes were collected by centrifugation of flotation for 6 h at 100 000 *g* in an SW 50.1 rotor (Beckman, Chaska, MN, USA). Then, precipitates were applied to carbon-coated grids that had been rendered hydrophilic by 0.1% (wt/wt) bacitracin. The grids were washed twice with 100 mm NH_4_ acetate/50 mm NH_4_HCO_3_ buffer (pH 7.4), negatively stained with 2% uranyl formate, and examined in JEM-2000EX TEM.

### Extraction of membrane constituent of *E. coli* and mass spectrum analysis

*E. coli* were pre-treated for 30 min at 4 °C by antisera or not. The membrane proteins were extracted as above, and then analyzed by SDS–PAGE. After stained with Coomassie Brilliant Blue R, four extra bands in the three treated groups were excised from gel and digested with trypsin. The treated samples were applied to the MALDI-TOF-TOF Analyzer (AB Sciex, Pte. Ltd., Singapore, Singapore). After tryptic peptide mass acquisition, mass fingerprinting searching was carried out in Swiss-Prot and NCBInr database using MASCOT (Matrix Science, Ltd., London, UK, http://www.matrixscience.com) online available software.

### Cloning of full-length *LPFP* cDNA and 3D structure prediction of protein

RNA was extracted from the liver of lamprey using the Catrimox-14 RNA Isolation Kit (TaKaRa, Kusatsu, Shiga, Japan) and converted to cDNA. To obtain the complete cDNA sequences, 3′- and 5′-RACE were performed using the GeneRacer Kit (Invitrogen, Waltham, MA, USA, 15596-018) with gene-specific primers. The amplified fragments were cloned into a pGEM-T easy vector and sequenced using an ABI PRISM 3730 DNA analyzer. The tertiary structure of LPFP was predicted by Phyre2 Protein Fold Recognition online server (http://www.sbg.bio.ic.ac.uk/phyre2/html/page.cgi?id=index) based on the template of zebrafish Dln1 (PDB 4ZNO) and analyzed by Swiss-PdbViewer.

### Sequence alignments and phylogenetic analyses

For constructing phylogenetic tree, a Neighbor-Joining (NJ) tree was constructed based on pairwise deletion of gaps/missing data with the MEGA version 5.1 and the p-distance matrix of amino acids model with 500 bootstrap replications.

### Preparation of recombinant protein

The full-length cDNA encoding LPFP was cloned into a pGEX-6P1 expression vector. The GST-tagged fusion protein was expressed in *E. coli* Rosetta (DE3) and purified by Glutathione-Sepharose affinity chromatography (GE Healthcare, Otelfingen, Switzerland).

### Sugar-binding assay of LPFP by Elisa

The sugar-binding assay was performed as described previously [17]. Briefly, 200 ng per well carbohydrates in 100 μl CBS buffer (pH 9.6) was coated the ELISA plate over 12 h at 4 °C respectively. Then the plates were blocked with 1% BSA in PBS. 100 μl LPFP was added into the plate by 0.5-log dilutions from 1 μM to 0.0079 μm and incubated at 37 °C for 1 h. The plate was then sequentially incubated with Rabbit-anti-LPFP primary antibody and HRP-conjugated goat anti-rabbit secondary antibody at 37 °C for 1 h. After each steps, the plate was washed with 0.5% tween-20 in PBS for 5 times. Finally, each well was incubated with 100 μl TMB for 15 min in the dark and stopped by 100 μl 2 m H_2_SO_4_ immediately. The absorbance was read at 450 nm.

### Oligomerization of LPFP

0.35 mgml^−1^ LPFP was incubated with 1.5 mgml^−1^ mannan (pH 6.0) at 25 °C for 1 h as described previously [17]. Then the protein was separated by SDS–PAGE and stained by coomassie blue.

### Preparation of polyclonal antibody against LPFP

To generate rabbit anti-LPFP polyclonal antibody, New Zealand rabbits were inoculated with the recombinant LPFP protein. Prior to inoculation, pre-immune serum was collected for control experiments. The animals were given multipoint intradermal injection for four times, each at 14 day intervals. Antisera were collected from each animal, and the polyclonal antibody in the serum was purified by protein G-affinity chromatography (GE Healthcare). The concentration of the purified antibody was adjusted to 1 mg ml^−1^ and stored at −20 °C in 50% glycerol. The titer and specificity of the purified antibody were monitored by ELISA and western blot assays (Supplementary Figure S7).

### Quantitative real-time PCR analysis of *LPFP* expression

RNAs from lamprey intestine, gill, leukocyte, liver, heart, skin, and kidney were extracted using the Catrimox-14 RNA Isolation Kit and converted to the corresponding cDNAs. Quantitative real-time PCR was performed in a LightCycler 480 real-time PCR system (Roche, Indianapolis, IN, USA) using GoTaq qPCR Master Mix kit (Promega, Madison, WI, USA, A6002). Forty-five PCR cycles were run, and the melting curve was recorded. The cycle threshold (Ct) values were obtained. The relative expression of *LPFP* mRNA in different tissues was calculated using 2^−∆CT^, in which ∆Ct=Ct (*LPFP*)–Ct (*GAPDH*). All tissues samples were performed in triplicates. The each kind of tissues was acquired from six lampreys, three lampreys were stimulated with LPS in 0.9% NaCl and three lampreys were simultaneously injected with 0.9% NaCl as control.

### Laser scanning confocal microscopy

To detect the deposition of LPFP on the surface of the target cells, HeLa cells were treated with 2% antisera for 30 min at 4 °C and then washed three times with PBS. The cells were fixed with 4% polyformaldehyde in PBS for 15 min at room temperature, washed three times with PBS, and then blocked with 3% BSA in PBS for 30 min at room temperature. Subsequently, the cells were incubated with 1 μg ml^−1^ rabbit anti-LPFP polyclonal Ab at 4 °C for 16 h, and stained with FITC-labeled goat anti-rabbit IgG (Cell Signaling Technology, Danvers, MA, USA, 2947). The cells were stained with DAPI for 5 min and then washed with PBS. After that they were photographed with a Carl Zeiss Axiovision 4 microscope (Carl Zeiss, Dublin, CA, USA).

### Depletion and replenishment of LPFP in lamprey antisera

To deplete LPFP in the lamprey antisera, 10 μl of HeLa cell-stimulated lamprey antisera was incubated with 0.25, 0.5, or 1 μgml^−1^ of LPFP antibody at 4 °C for 4 h. Subsequently, 20 μl protein G agarose (Roche) was added to the mixture and incubated at 4 °C for 4 h followed by centrifugation at 200 *g* for 10 min. The supernatant was collected for cytotoxic analysis, and the precipitate was analyzed by western blot. To restore the cytotoxicity of the LPFP-depleted antisera, purified recombinant LPFP was added to the LPFP-depleted antisera to a final concentration of 0.25, 0.5, 1 or 2 μgml^−1^, and the cytotoxic activity of the antisera was determined as described.

### Depletion of VLR and C3 protein and Western blot assay

To deplete VLR or C3 protein in the antisera, 4 μl of the antisera was incubated with 10 μg of anti-VLR monoclonal antibody (mAb) or anti-C3 polyclonal antibody (pAb) at 4 °C for overnight. Protein G agarose was then added to the sample followed by incubation at 4 °C for 4 h. After centrifugation, the supernatant was collected. To perform Western blot assay, HeLa cells were incubated with VLR-depleted or C3-depleted antisera for 30 min at 4 °C, and the sample was then centrifuged at 12 000×*g* for 30 min, and the precipitate was resuspended in loading buffer and subjected to 10% SDS–PAGE followed by Western blot analysis. The deposition of C3 and LPFP on the surface of HeLa cells was detected with anti-C3 and anti-LPFP rabbit pAb respectively. Briefly, the proteins in the gel were transferred to PVDF membrane (Millipore, Billerica, MA, USA) after SDS–PAGE. The membrane was first incubated with anti-C3 or anti-LPFP antibody, and then with goat anti-rabbit IgG conjugated to horseradish peroxidase, and positive signal was detected by the enhanced chemiluminescence reagent (Pierce, Waltham, MA, USA, 23225).

### Statistical analysis

All experiments were performed at least three times. Determination of statistical differences was performed with Prism 6 (Graphpad Software, Inc., San Diego, CA, USA) using *t*-tests (to compare two groups with similar variances), or one-way ANOVA with Turkey’s multiple comparison test (to compare more than two groups). Error bars presented as the means±s.d.

## References

[bib1] Serna M, Giles JL, Morgan BP, Bubeck D. Structural basis of complement membrane attack complex formation. Nat Commun 2016; 7: 10587. 26841837 10.1038/ncomms10587 PMC4743022

[bib2] Dudkina NV, Spicer BA, Reboul CF et al. Structure of the poly-C9 component of the complement membrane attack complex. Nat Commun 2016; 7: 10588. 26841934 10.1038/ncomms10588 PMC4742998

[bib3] Pancer Z, Amemlya CT, Ehrhardt GRA et al. Somatic diversification of variable lymphocyte receptors in the agnathan sea lamprey. Nature 2004; 430: 174–180. 15241406 10.1038/nature02740

[bib4] Han BW, Herrin BR, Cooper MD, Wilson IA. Antigen recognition by variable lymphocyte receptors. Science 2008; 321: 1834–1837. 18818359 10.1126/science.1162484 PMC2581502

[bib5] Wu F, Chen L, Liu X et al. Lamprey variable lymphocyte receptors mediate complement-dependent cytotoxicity. J Immunol 2013; 190: 922–930. 23293356 10.4049/jimmunol.1200876

[bib6] Nonaka M, Kimura A. Genomic view of the evolution of the complement system. Immunogenetics 2006; 58: 701–713. 16896831 10.1007/s00251-006-0142-1 PMC2480602

[bib7] Nonaka M, Fujii T, Kaidoh T et al. Purification of a lamprey complement protein homologous to the third component of the mammalian complement system. J Immunol 1984; 133: 3242–3249. 6491286

[bib8] Nonaka M, Takahashi M. Complete complementary DNA sequence of the third component of complement of lamprey. Implication for the evolution of thioester containing proteins. J Immunol 1992; 148: 3290–3295. 1578150

[bib9] Matsushita M, Matsushita A, Endo Y et al. Origin of the classical complement pathway: Lamprey orthologue of mammalian C1q acts as a lectin. Proc Natl Acad Sci USA 2004; 101: 10127–10131. 15218103 10.1073/pnas.0402180101 PMC454176

[bib10] Muller-Eberhard HJ. The membrane attack complex of complement. Annu Rev Immunol 1986; 4: 503–528. 3518749 10.1146/annurev.iy.04.040186.002443

[bib11] Young JD, Cohn ZA, Podack ER. The ninth component of complement and the pore-forming protein (perforin 1) from cytotoxic T cells: structural, immunological, and functional similarities. Science 1986; 233: 184–190. 2425429 10.1126/science.2425429

[bib12] Law RH, Lukoyanova N, Voskoboinik I et al. The structural basis for membrane binding and pore formation by lymphocyte perforin. Nature 2010; 468: 447–451. 21037563 10.1038/nature09518

[bib13] Podack ER, Hengartner H, Lichtenheld MG. A central role of perforin in cytolysis? Annu Rev Immunol 1991; 9: 129–157. 1910674 10.1146/annurev.iy.09.040191.001021

[bib14] Dang TX, Hotze EM, Rouiller I, Tweten RK, Wilson-Kubalek EM. Prepore to pore transition of a cholesterol-dependent cytolysin visualized by electron microscopy. J Struct Biol 2005; 150: 100–108. 15797734 10.1016/j.jsb.2005.02.003

[bib15] Xue Z, Liu X, Pang Y et al. Characterization, phylogenetic analysis and cDNA cloning of natterin-like gene from the blood of lamprey, Lampetra japonica. Immunol Lett 2012; 148: 1–10. 22914553 10.1016/j.imlet.2012.08.005

[bib16] Magalhaes GS, Lopes-Ferreira M, Junqueira-de-Azevedo IL et al. Natterins, a new class of proteins with kininogenase activity characterized from Thalassophryne nattereri fish venom. Biochimie 2005; 87: 687–699. 16054523 10.1016/j.biochi.2005.03.016

[bib17] Jia N, Liu N, Cheng W et al. Structural basis for receptor recognition and pore formation of a zebrafish aerolysin-like protein. EMBO Rep 2016; 17: 235–248. 26711430 10.15252/embr.201540851 PMC5290818

[bib18] Meagher JL, Winter HC, Ezell P, Goldstein IJ, Stuckey JA. Crystal structure of banana lectin reveals a novel second sugar binding site. Glycobiology 2005; 15: 1033–1042. 15944373 10.1093/glycob/cwi088

[bib19] Kanagawa M, Liu Y, Hanashima S et al. Structural basis for multiple sugar recognition of Jacalin-related human ZG16p lectin. J Biol Chem 2014; 289: 16954–16965. 24790092 10.1074/jbc.M113.539114 PMC4059138

[bib20] Mancheno JM, Tateno H, Goldstein IJ, Martinez-Ripoll M, Hermoso JA. Structural analysis of the Laetiporus sulphureus hemolytic pore-forming lectin in complex with sugars. J Biol Chem 2005; 280: 17251–17259. 15687495 10.1074/jbc.M413933200

[bib21] Parker MW, Buckley JT, Postma JP et al. Structure of the Aeromonas toxin proaerolysin in its water-soluble and membrane-channel states. Nature 1994; 367: 292–295. 7510043 10.1038/367292a0

[bib22] Dunkelberger JR, Song WC. Complement and its role in innate and adaptive immune responses. Cell Res 2010; 20: 34–50. 20010915 10.1038/cr.2009.139

[bib23] Oettinger MA, Schatz DG, Gorka C, Baltimore D. RAG-1 and RAG-2, adjacent genes that synergistically activate V(D)J recombination. Science 1990; 248: 1517–1523. 2360047 10.1126/science.2360047

[bib24] Sahu A, Lambris JD. Structure and biology of complement protein C3, a connecting link between innate and acquired immunity. Immunol Rev 2001; 180: 35–48. 11414361 10.1034/j.1600-065x.2001.1800103.x

[bib25] Podack ER, Kolb WP, Muller-Eberhard HJ. The C5b-9 complex: subunit composition of the classical and alternative pathway-generated complex. J Immunol 1976; 116: 1431–1434. 1270802

