# Supplementary material for: A pore-forming protein implements VLR-activated complement cytotoxicity in lamprey
Source: Cell Discov. 2017 Sep 19;3:17033–. doi: 10.1038/celldisc.2017.33 (PMC5605768; doi:10.1038/celldisc.2017.33)

Supplementary figure legends

Supplementary Figure S1 Comparison of morphological changes of the HeLa cells killed by lamprey antisera or mouse antisera at indicated times. Hela cells were cultured in 96-well plate and incubated with lamprey antisera or mouse antisera respectively. Images were collected at 0 h, 3 h, 6 h and 24 h.

Supplementary Figure S2 TEM of membrane extracts from RRBCs, *E. coli* cells and HeLa cells. No pore-like structures observed in the membrane proteins of all three types of cells. Scale bar, 20 nm.

Supplementary Figure S3 Full length cDNA of LPFP. The full length cDNA of LPFP consisted of 1189 bp, with a 942-bp ORF.

Supplementary Figure S4 Amino acid sequences of LPFP. The solid line indicates the jacalin-like lectin domain and the dotted line indicates the pore-forming domain.

Supplementary Figure S5 Sequence alignment of LPFP and Dln1 by GeneDoc. The identical amino acid residues are highlighted in black.

Supplementary Figure S6 Phylogenetic analysis of lectin domain and pore-forming domain of lamprey LPFP. (a) Neighbor-Joining tree of lectin domain of lamprey LPFP with other natterin-like proteins was constructed with the MEGA version 5.1 using the full-length sequence. (b) Neighbor-Joining tree of LPFP with other lectinswas constructed as in (a). (c) Neighbor-Joining tree of LPFP with other proteins pore-forming domain from other species was constructed as in (a). The numbers at the nodes indicate bootstrap values.

Supplementary Figure S7 Preparation of recombinant protein and polyclonal antibody of LPFP. (a) PCR product of full-length *LPFP*. (b) Purified recombinant

protein of full-length LPFP. (c) Purified rabbit anti-LPFP polyclonal antibody. (d) Titer of rabbit anti-LPFP polyclonal antibody by ELISA assay. (e) Specificity of rabbit anti-LPFP polyclonal antibody against LPFP in lamprey serum detected by Western blot assay. Serum was from three fishes. Anti-LPFP polyclonal antibodies were diluted from 1:1000 to 1:8000.

Supplementary Figure S1

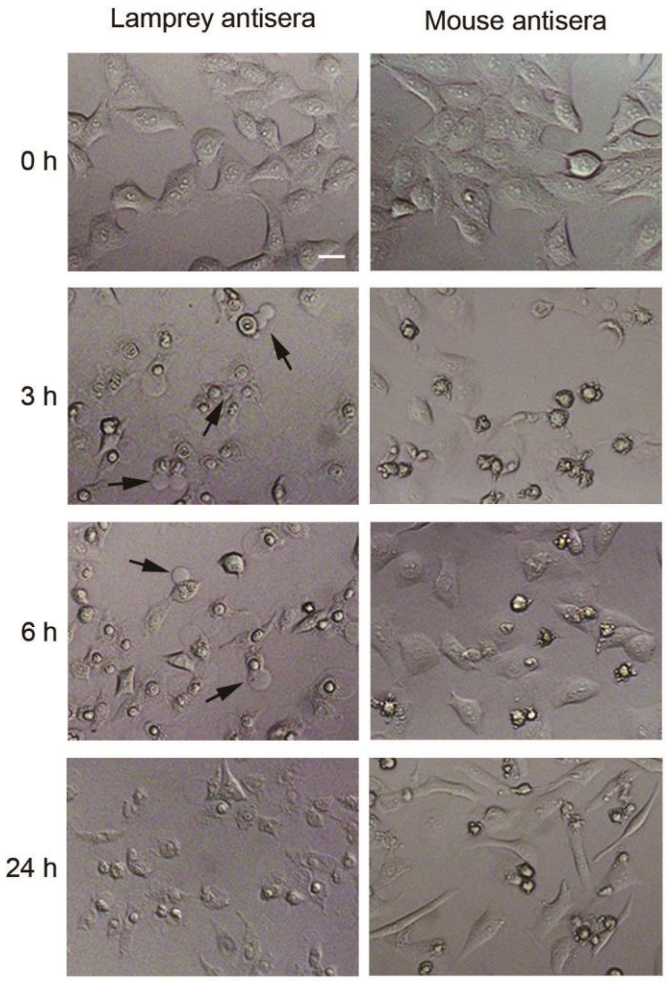

Supplementary Figure S2

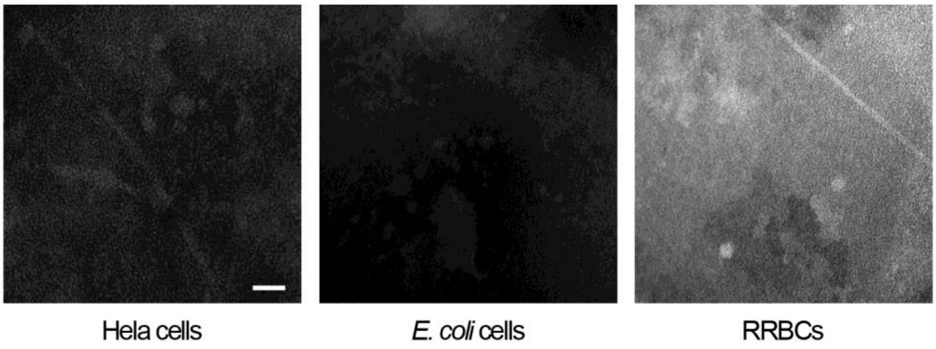

Supplementary Figure S3

50 CTAATACGAC TCACTATAGG GCAAGCAGTG GTATCAACGC AGAGTACATG

100 GGGACATCTG CTCTTCGCGG AGTCGACTGC TTTCACGTGA GTGGTCGAAA

150 CCATGGTGTA CCCGACCACA CTTCACATCA TTGGCGGCCA AGGTGGAAAC

200 GCGTTCTCGT TCAACGGGCA GGAGAATGCG GCGACGCTGC AGAAGCTCTC

250 TGTGAGCGTT GGGGGATGGC AGGTGAGGGG CGTGCAGGTG TGGCTGACGG

300 ACGGGCGCAG GGAGACATTC GGCGCCATGG ACTCCTCCGC TAAGGAGTTC

350 GAATTCGAGT CGGGCGAGTT CATCAAGAGC CTCTCGCTGT GGGGCAACGG

400 AGCCGGCACT CGCCTGGGCG CTATCAAGTT CATAACGAGC CGCAGCCGCG

450 AGTTCTTTGC CAAGATGACG GACTGGGGGC TCAAGACCGA GTACAAGATC

500 GACGTGGGCT CTGGCATCTG CCTGGGTGTT CAGGGCCGAG GGGGGTCCGA

550 CATTGACTCC ATGGGCTTCA TCTTCATCAA TGCCATAAAA TCGTCGGTGA

600 TCCAGGACAT GAAGTACCCG ACCATGCACC AAATTCTGCC CAACGTGCAG

650 ATGGAGGAGA ACAAAGAAAT GGAGTACAAG AACGACACCA GCATCGTGCA

700 ATCGTACACA TTCGAGAGCT CCAAGAAGAT CATTAAAAAG TCATCGTGGT

750 CCACCACCAA CAAGATCGAG TCCACCTTCA GCCTGTCGGT GAAGGCCGGC

800 ATCCCCGAGG TCATGGAGGT GGAGACCGGA TTCAGCTTCA CCGTGGGCAG

850 TGAGAGCACG CACGCGGTGG AGGAGTCCGA GGAGAAGACG GAAACGCTCA

900 CGTTCCCCGT CACTGTCCCG ACGCACAAGA CCGTCACCGT GGTCGCCAAC

950 ATCGGGCGCG CCGACATCGA CCTTCCGTAC ACGGCCCTGC TGCGCATCAC

1000 CTGCGTGAAC GGCGCATCCC TTGACGCTCC CCTGAGCGGC ATCTACAAGG

1050 GGCTCACCTA CACCAAGATG ACCGCCGTTG CTACCGAGAG CTAGAGCGGC

1100 GTTTCGCAGC ACCCCTTTTT TTTAATTGGT GATGTTGATG AAGTGTTCTA

1150 AAGGAAAGTG CTTCTTTGGC TGTGTAAAAT TCACCAATAA AAAAGGCCTA

1189 ACATTGAAAA AAAAAAAAAA AAAAAA

Supplementary Figure S4

MVYPTTLHIIGGQGGNAFSFNGQENAATLQKLSVSVGGWQVRGVQVWLTD
GRRETFGAMDSSAKEFEFESGEFIKSLSLWGNGAGTRLGAIKFITSRSRE
FFAKMTDWGLKTEYKIDVGSGICLGVQGRGGSDIDSMGFIFINAIKSSVI
QDMKYPTMHQILPNVQMEEIKEMEYKNDTSIVQSYTFESSKKIIKKSSWS
TTNKIESTFSLSVKAGIPEVMEVETGFSFTVGSESTHAVEESEEKTETLT
FPVTVPTHKTVTVVANIGRADIDLPYTALLRITCMNGASLDAPLSGIYKG
LTYTKMTAVATES

Supplementary Figure S5

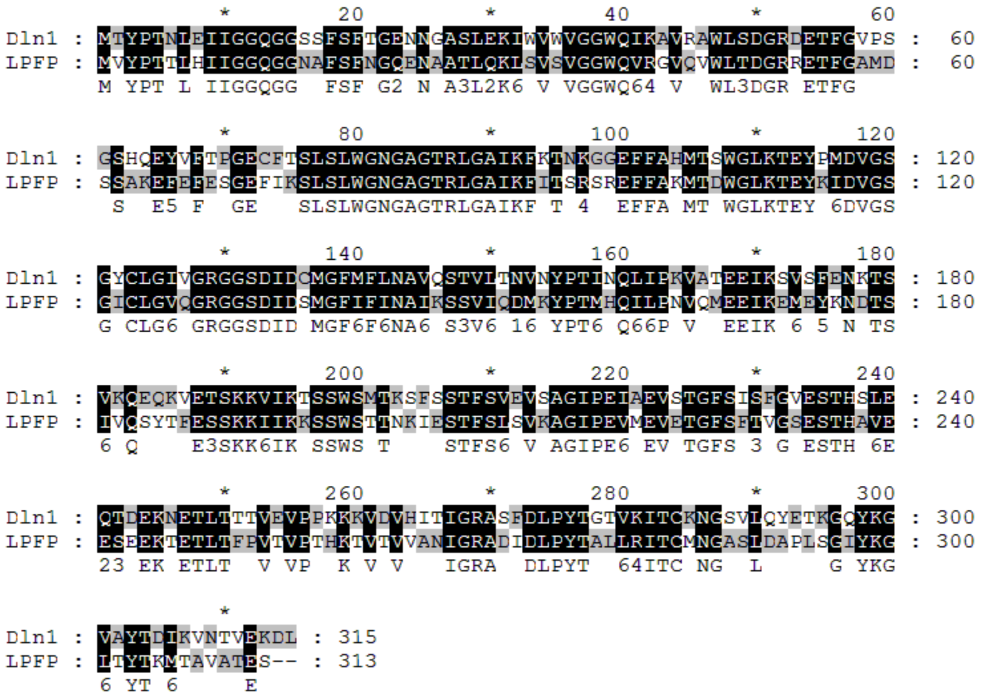

Supplementary Figure S6

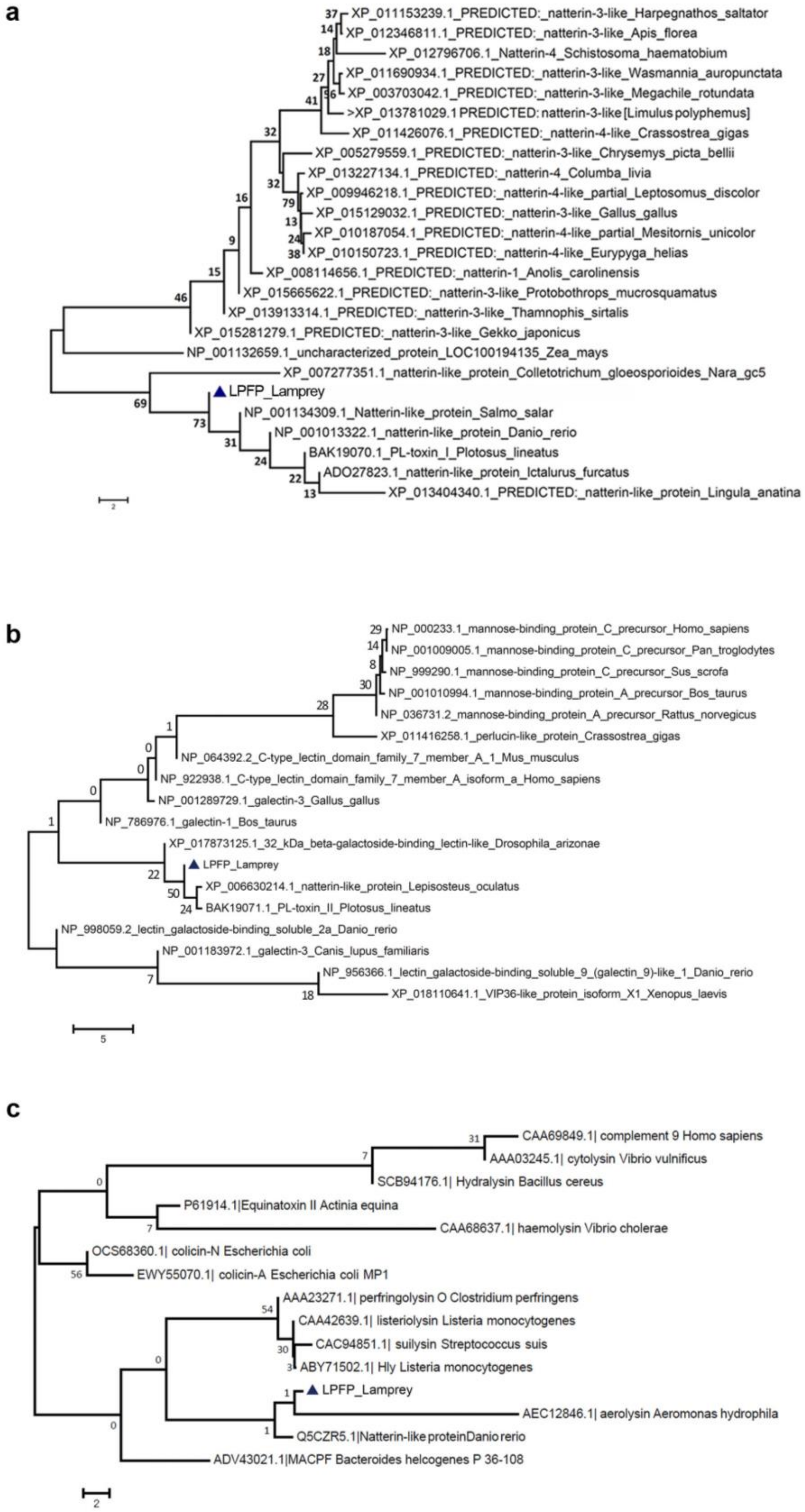

Supplementary Figure S7

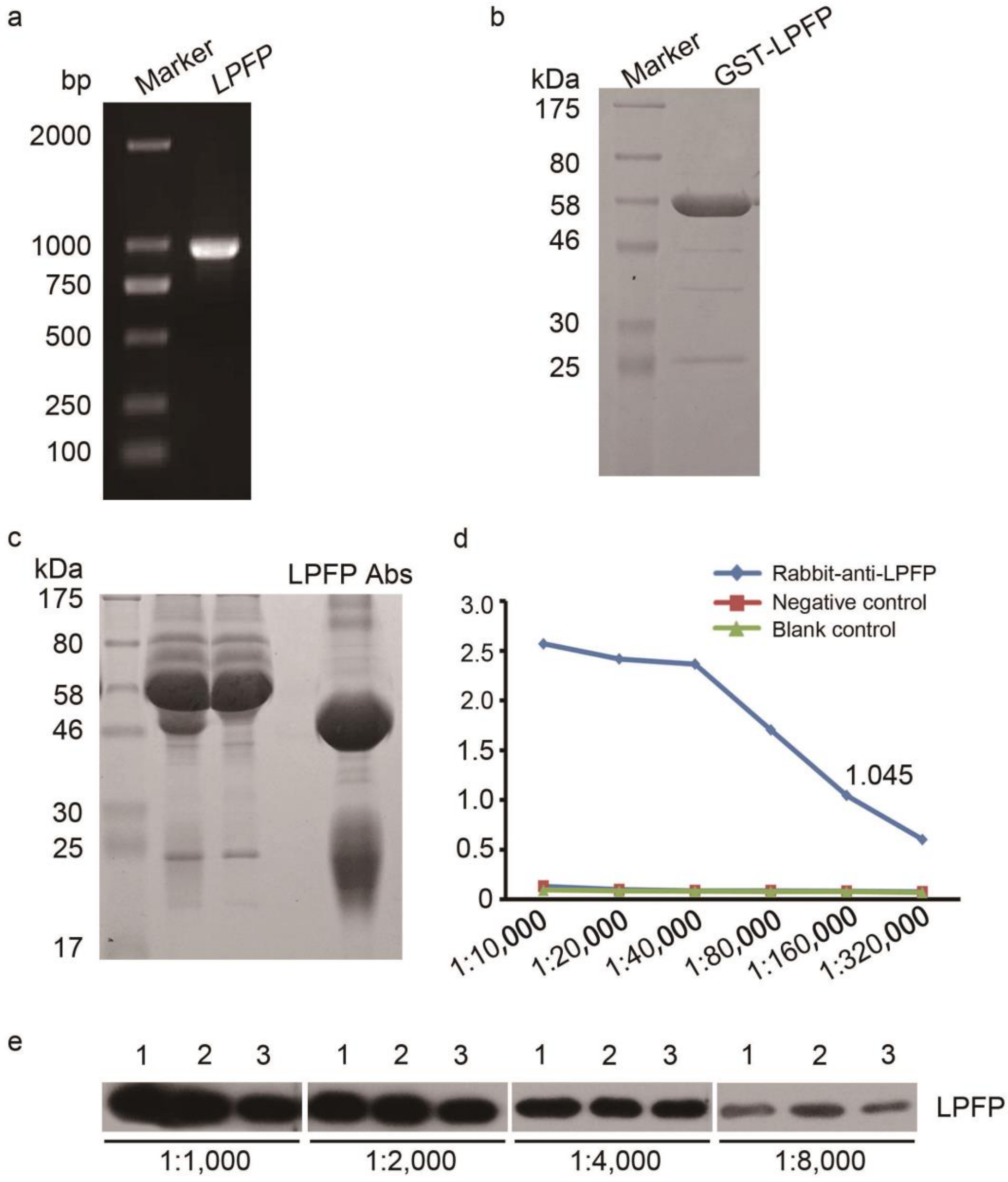

Supplement: Supplementary information [file celldisc201733-s1.pdf]
